# Kinetics and intracellular compartmentalization of HTLV-1 gene expression

**DOI:** 10.1186/1742-4690-8-S1-A204

**Published:** 2011-06-06

**Authors:** Francesca Rende, Ilaria Cavallari, Alberto Corradin, Micol Silic-Benussi, Frederic Toulza, Gianna M Toffolo, Yuetsu Tanaka, Steven Jacobson, Graham P Taylor, Donna M D'Agostino, Charles R M Bangham, Vincenzo Ciminale

**Affiliations:** 1 Department of Oncology and Surgical Sciences, University of Padova, Padova, I-35128, Italy; 2 Istituto OncologicoVeneto–IRCCS, Padova, Italy; 3 Department of Information Engineering, University of Padova, Padova, Italy; 4 Department of Immunology, Imperial College, London, UK; 5 Department of Immunology, Graduate School of Medicine, University of the Ryukyus, Okinawa, Japan; 6 Viral Immunology Section, Neuroimmunology Branch, National Institutes of Health, Bethesda, MD, USA

## 

Human T cell leukemia virus Type 1 (HTLV-1) codes for at least 9 alternatively-spliced transcripts and two major regulatory proteins named Tax and Rex that function at the transcriptional and post-transcriptional levels, respectively. We investigated the temporal sequence of HTLV-1 gene expression in primary cells from infected patients using splice site-specific quantitative RT-PCR. The results indicated a two-phase kinetics with the Tax/Rex mRNA preceding expression of other viral transcripts. Using transfection of HTLV-1 molecular clones and subcellular RNA fractionation we demonstrated the Rex-dependency of the two-phase kinetics and determined the compartmentalization of the individual mRNAs, showing that over 90% of the HBZ mRNAs were retained in the nucleus. Mathematical modelling revealed the importance of a functional delay of Rex function compared to Tax, which was supported by experimental evidence of delayed accumulation and longer half-life of Rex. These data provided the first evidence for a temporal pattern of HTLV-1 expression and revealed major differences in the intracellular compartmentalization of HTLV-1 transcripts.

